# Serum IgG1 and IgG3 Antibody Responses to *Chlamydia trachomatis* Pgp3 and Hsp60 in Tubal Factor Infertility

**DOI:** 10.1093/infdis/jiaf092

**Published:** 2025-02-21

**Authors:** Tiina Holster, Päivi Joki-Korpela, Hong Yu, Robert C Brunham, Aila Tiitinen, Jorma Paavonen, Mirja Puolakkainen

**Affiliations:** Department of Obstetrics and Gynecology, Reproductive Medicine Unit, University of Helsinki and Helsinki University Hospital, Helsinki, Finland; University of Helsinki, Helsinki, Finland; University of Helsinki, Helsinki, Finland; Ovumia Helsinki, Helsinki, Finland; Department of Medicine, British Columbia Centre for Disease Control, University of British Columbia, Vancouver, British Columbia, Canada; Department of Medicine, British Columbia Centre for Disease Control, University of British Columbia, Vancouver, British Columbia, Canada; University of Helsinki, Helsinki, Finland; University of Helsinki, Helsinki, Finland; University of Helsinki, Helsinki, Finland; Virology and Immunology, University of Helsinki and Helsinki University Hospital, Helsinki, Finland

**Keywords:** *Chlamydia trachomatis*, tubal factor infertility, serology, Pgp3, Hsp60

## Abstract

**Background:**

Our goal was to investigate IgG1 and IgG3 antibody responses to *Chlamydia trachomatis* proteins Pgp3 and Hsp60 in women with tubal factor infertility (TFI). Our goal was to determine the role of these biomarkers in the diagnosis of *C. trachomatis*-associated TFI, and assess their sensitivity and specificity for detecting tubal pathology.

**Methods:**

Serum samples were collected from 258 subfertile women, and 34 women positive for *C. trachomatis* by nucleic acid amplification test (NAAT). IgG1 and IgG3 antibodies to Pgp3 and Hsp60 were measured using enzyme immune assays.

**Results:**

Pgp3 IgG1 antibodies were detected in 68.2% of TFI cases and 31.8% of controls (non-TFI), while Hsp60 IgG1 antibodies were found in 36.4% of TFI cases. Pgp3 IgG1 had the highest sensitivity for TFI (68.2%; 95% confidence interval [CI], 45.1%–86.1%), while Hsp60 IgG3 was the most specific (93.6%; 95% CI, 89.7–96.4). Antibody levels increased with tubal damage severity. Among the 34 NAAT-positive women, 78.8% were positive for Pgp3 IgG1.

**Conclusions:**

Pgp3 IgG1 antibody was a sensitive marker for detecting *C. trachomatis*-related TFI, while Hsp60 IgG3 antibody was highly specific. These findings suggest that Pgp3 and Hsp60 antibodies and antibody subclass testing may be useful diagnostic tools for assessing TFI.

## Teaser

The study examined IgG1 and IgG3 antibodies to *Chlamydia trachomatis* proteins Pgp3 and Hsp60 in women with tubal factor infertility. Results indicated Pgp3 IgG1 is a sensitive marker and Hsp60 IgG3 is a specific marker for diagnosing *Chlamydia*-associated TFI.

*Chlamydia trachomatis* is the leading cause of bacterial sexually transmitted infections globally. If untreated, *C. trachomatis* infection can progress from lower genitourinary infection to pelvic inflammatory disease (PID). PID can also be caused by sexually transmitted pathogens other than *C. trachomatis*, by microbes colonizing vagina, or by pathogens in other body sites [1]. Approximately 20% of PID cases are due to *C. trachomatis* [2]. PID can result in scarring and obstruction of the fallopian tubes leading to tubal factor infertility (TFI). Estimating the population-level risk of TFI following *C. trachomatis* infection is challenging. The estimated risk of developing TFI after an episode of chlamydial salpingitis is 6.9% [2].

Because TFI occurs after the initial infection has resolved [1] and self-reported history of infection is unreliable [3], serology remains the best way to evaluate history of *C. trachomatis* infection. Positive serology has been linked to TFI, although assay performance varies depending on the antigen used [4–7].

Recent studies have highlighted the potential role of Pgp3 immunoglobulin G (IgG) antibody for monitoring *C. trachomatis* infection sequelae [8, 9]. Pgp3 is a plasmid-encoded, highly immunogenic protein [10–12]. An anti-Pgp3 response indicates ongoing or past *C. trachomatis* infection [10–12]. *C. trachomatis* also codes for Hsp60, a heat shock protein 60, which has been implicated in the immunopathogenesis of chlamydial infection [13, 14]. However, its role as a principal driver of immunopathology, including scarring, has recently been questioned and infected epithelial cells recruiting immune cells are suggested as a stimulus for fibrotic immunopathology [15]. Hsp60 may facilitate fibrosis indirectly as an inducer of inflammation, and antibody to Hsp60 reflects tissue damage due to chronic infection and associated inflammation [15].

A potential concern in chlamydial serology is cross-reactivity with other *Chlamydia* species, such as *Chlamydia pneumoniae* [16]. This complicates evaluation of the biomarkers if whole bacteria, antigens present in genus *Chlamydia* (ompA, ompB), or antigens shared among other microbes (Hsp60) are used as antigens. Careful set up, execution, and interpretation of the assays can diminish the confounding effect. As an example, Pgp3 is highly conserved in *C. trachomatis* and not present in *C. pneumoniae* [10, 11, 17]. Furthermore, Pgp3 assays demonstrate high sensitivity and specificity and Pgp3 has proven superior to many other antigens used in chlamydial serology [11, 18, 19].

Most *C. trachomatis* serologic assays are limited by low to moderate sensitivity. Geisler et al developed an IgG1- and IgG3-based enzyme-linked immunosorbent assay (ELISA) with excellent sensitivity and specificity for detecting anti-EB antibody responses [20]. Yu et al later compared this IgG1 ELISA to conventional total IgG ELISA using various *C. trachomatis* antigens, including Pgp3 and Hsp60. Their results confirmed the superior sensitivity and likely higher specificity of the IgG1 ELISA, demonstrated by significantly higher optical density (OD) values in *C. trachomatis*-seropositive sera and markedly lower background noise in sera from *C. trachomatis*-naive women compared to the total IgG ELISA [12].

IgG1 and IgG3 are the predominant human serum antibody subclasses formed against *C. trachomatis* infection [20]. IgG1 contributes to both the early and late phases of the immune response, whereas IgG3 typically dominates the early phase but declines more rapidly than IgG1. Despite this, IgG3 is potentially the most proinflammatory human IgG subclass due to its strong effector functions, such as complement activation and binding to Fc receptors on immune cells [21]. A study by Steiner et al found that IgG3 seropositivity to *C. trachomatis* is associated with a reduced likelihood of pregnancy, even when the fallopian tubes remain open. This finding suggests that IgG3 testing may aid in diagnosing TFI [22]. In this study, we wanted to investigate IgG1 and IgG3 antibody responses to *C. trachomatis* Pgp3 and Hsp60 among women with TFI to better understand the role of these biomarkers in the diagnosis of *C. trachomatis*-related TFI.

## METHODS

### Study Population

Serum specimens were collected from 258 unselected subfertile women referred for infertility to the Helsinki University Hospital between July 2007 and December 2010 [23]. In brief, women were eligible if they had failed to achieve pregnancy after 12 months or more of regular unprotected intercourse, and were under 40 year of age. TFI was defined as an occlusion of at least 1 fallopian tube by hysterosonosalpingography or laparoscopy. Controls were subfertile women with no tubal pathology.

Also, sera from 34 *C. trachomatis* nucleic acid amplification test (NAAT)-positive women attending the outpatient sexually transmitted infection clinic of the Helsinki University Hospital, Finland during 2009–2011 [24] were studied. The patients visited the clinic because of symptoms, for follow-up, or because of notification by an infected partner. Swabs for NAAT and sera samples were collected at the same visit.

All procedures performed in studies involving human participants were in accordance with the ethical standards of the institutional research committee and with the 1964 Helsinki declaration and its later amendments. The study was approved by the Helsinki University Hospital Ethical Committee (Dnro 29/E9/07). Informed consent was obtained from all women.

### Serology

Serum samples from the subfertile women were collected at the first outpatient clinic visit and stored at −20°C until analyzed. IgG1 and IgG3 antibody responses to *C. trachomatis* Pgp3 and Hsp60 were analyzed by enzyme immune assay (EIA). The sera had earlier been studied by microimmunofluorescence test (MIF), and EIA using *C. trachomatis* major outer membrane protein (MOMP) TroA and HtrA as antigens [5, 23].

The purified recombinant Pgp3 and Hsp60 proteins were obtained from Biomatik [12]. The proteins were diluted in 0.1 M carbonate–bicarbonate buffer, pH 9.6 (Sigma-Aldrich), and 100 ng of the protein was coated onto flat-bottomed polystyrene microtiter plates (Costa Assay 96-well plate; Corning). After coating overnight at 4°C, the wells were washed 3 times with 1 × phosphate-buffered saline plus 0.05% Tween20 (PBST) and blocked at 37°C with 3% bovine serum albumin (BSA) in 1 × PBS for 2 hours. Wells were again washed 2 times with PBST, and 100 μL of sera diluted 1:32 in 0.5% BSA in 1 × PBS were applied to wells in triplicate, and incubated overnight at 4°C. After 4 washes with PBST, 100 μL of the 1:500 diluted alkaline phosphatase (AP)-conjugated anti-human immunoglobulin G (IgG) secondary antibodies were added per well and incubated at 37°C for 2 hours. To test for IgG1 antibodies, mouse anti-human IgG1, Fc fragment specific alkaline phosphatase conjugate (HP6069; Millipore Sigma) and mouse anti-human IgG1 hinge-AP (4E3; Southern Biotech) were pooled. To test for IgG3 antibodies, mouse anti-human IgG3 hinge-AP (HP6050; Southern Biotech) was used. Finally, the plates were again washed 4 times with PBST, and 100 μL/well of *p*-NPP (1 tablet of *p*-nitrophenyl phosphate plus 1 tablet of TRIS in 20 mL H_2_O; Sigma) was added and incubated at room temperature in the dark. The optical density at 405 nm was read at exactly 30 minutes after addition of substrate. The cutoff values were based on the absorbance values (mean + 2 SD) obtained using specimens of individuals with no antibody detectable by MIF (n = 127). The cutoff for Pgp3 IgG1 was 0.263 and for IgG3 0.140. The cutoff for HSP60 IgG1 was 0.262 and for IgG3 0.145.

### Statistical Analysis

The χ^2^ test was used for the analysis of categorical data, and continuous variables were compared by Mann-Whitney *U* test and Kruskal-Wallis test. To evaluate the performance of Pgp3 and Hsp60 antibodies for TFI, we calculated sensitivities, specificities, positive predictive values, negative predictive values, positive likelihood ratios, and negative likelihood ratios with 95% confidence intervals (CI). Statistical analysis was performed by IBM SPSS Statistics 29.0.

## RESULTS

The demographics of the subfertile women have been described earlier [23]. Of the 258 women, 22 (8.5%) had TFI. Bilateral tubal occlusion was found in 5 women and unilateral tubal occlusion in 17 women. At the time of serum sampling, none of the subfertile women had cervical *C. trachomatis* by NAAT.

Self-reported history of chlamydial infection was associated with the presence of serum Pgp3 IgG1 (70.6% vs 32.6%, *P* = .002). The baseline characteristics by the presence of IgG1 and IgG3 antibodies to Pgp3 and Hsp60 are presented in Table 1.

**Table 1.** Baseline Characteristics by the Presence of IgG1 and IgG3 Antibodies to Pgp3 and Hsp60

Variable	Pgp3 IgG1 Positive (n = 90)	Pgp3 IgG1 Negative (n = 168)	*P* Value	Pgp3 IgG3 Positive (n = 44)	Pgp3 IgG3 Negative (n = 214)	*P* Value	Hsp60 IgG1 Positive (n = 48)	Hsp60 IgG1 Negative (n = 210)	*P* Value	Hsp60 IgG3 Positive (n = 20)	Hsp60 IgG3 Negative (n = 238)	*P* Value
Age, y, mean (SD), range	31.2 (4.3), 21–39	31.4 (4.1), 21–40	.62	31.5 (4.3), 21–39	31.3 (4.1), 21–40	.84	31.5 (4.7), 21–40	31.3 (4.0), 21–39	.76	29.9 (4.1), 22–37	31.4 (4.1), 21–40	.16
Type of infertility, n (%)			.2			.004			.18			.65
Primary (n = 182)	59 (65.6)	123 (73.2)		23 (52.3)	159 (74.3)		30 (62.5)	152 (72.4)		15 (75.0)	167 (70.2)	
Secondary (n = 76)	31 (34.4)	45 (26.8)		21 (47.7)	55 (25.7)		18 (37.5)	58 (27.6)		5 (25.0)	71 (29.8)	
Cause of infertility, n (%)												
Endometriosis (n = 37)	11 (12.2)	26 (15.5)	.48	6 (13.6)	31 (14.5)	.88	9 (18.8.)	28 (13.3)	.33	2 (10.0)	35 (14.7)	.75
Male factor (n = 29)	8 (8.9)	21 (12.5)	.38	3 (6.8)	26 (12.1)	.43	4 (8.3)	25 (11.9)	.62	1 (5.0)	28 (11.8)	.71
Tubal factor (n = 22)	15 (16.7)	7 (4.2)	**<.001**	9 (20.9)	13 (6.1)	.**002**	8 (16.7)	14 (6.7)	.**03**	5 (25.0)	17 (7.1)	.**006**
Ovulatory disorder (n = 56)	24 (26.7)	32 (19.0)	.16	11 (25.0)	45 (21.0)	.56	13 (27.1)	43 (20.5)	.32	6 (30.0)	50 (21.0)	.35
Unexplained infertility (n = 99)	27 (30.0)	72 (42.9)	.04	15 (34.1)	84 (39.3)	.52	12 (25.0)	87 (41.4)	.04	7 (35.0)	92 (38.7)	.75
Prior extrauterine pregnancy, n (%)			.19			.27			.08			.07
Yes (n = 6)	4 (4.4)	2 (1.2)		2 (4.5)	4 (1.9)		3 (6.3)	3 (1.4)		2 (10.0)	4 (1.7)	
No (n = 252)	86 (95.6)	166 (98.8)		42 (95.5)	210 (98.1)		45 (93.8)	207 (98.6)		18 (90.0)	234 (98.3)	
Prior chlamydia, n (%)			.**001**			.16			.53			1.00
Yes (n = 17)	12 (13.3)	5 (3.0)		5 (11.4)	12 (5.6)		4 (8.3)	13 (6.2)		1 (5.0)	16 (6.7)	
No (n = 241)	78 (86.7)	163 (97.0)		39 (88.6)	202 (94.4)		44 (91.7)	197 (93.8)		19 (95.0)	222 (93.3)	
Smoking status, n (%)^a^			.06			.**001**			.31			.39
Yes (n = 45)	21 (24.1)	24 (14.5)		15 (34.9)	30 (14.4)		11 (22.9)	34 (16.7)		5 (25.0)	40 (17.2)	
No (n = 207)	66 (75.9)	141 (85.5)		28 (65.1)	179 (85.6)		37 (77.1)	170 (83.3)		15 (75.0)	192 (82.8)	

^a^Data is missing from 6 patients.

### *C. trachomatis* Pgp3 and Hsp60 IgG1 and IgG3 Antibodies in Subfertile Women

Of the 258 samples, 90 (32.5%) had *C. trachomatis* Pgp3 IgG1 antibodies, and 44 (15.9%) had Pgp3 IgG3 antibodies. Seropositivity to Pgp3 was more common among women with TFI, compared to women with non-TFI (68.2% vs 31.8%, *P* < .001 for IgG1; 40.9% vs 14.8%, *P* = .002 for IgG3). TFI was also associated with Hsp60 IgG1 antibody (36.4% vs 16.9%, *P* = .03) and IgG3 antibody (22.7% vs 6.4%, *P* = .006) (Table 2).

**Table 2.** The prevalence of *Chlamydia*  *trachomatis* Pgp3 and Hsp60 IgG Antibodies in Women With Tubal Factor Infertility (TFI) and Other Causes of Subfertility

Antibody	TFI, % (n = 22)	Non-TFI, % (n = 236)	*P* Value
Pgp3 IgG1 (n = 90)	68.2 (15/22)	31.8 (75/236)	<.001
Pgp3 IgG3 (n = 44)	40.9 (9/22)	14.8 (35/236)	.002
Hsp60 IgG1 (n = 48)	36.4 (8/22)	16.9 (40/236)	.03
Hsp60 IgG3 (n = 20)	22.7 (5/22)	6.4 (15/236)	.006

Values in parentheses explain the number of individuals with a positive finding/number of individuals with TFI.

**Figure 1.**
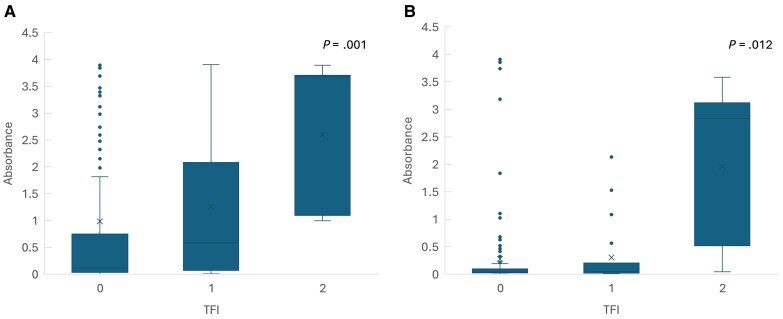
The absorbance values of *Chlamydia trachomatis* Pgp3 IgG1 (*A*) and IgG3 (*B*) antibody by the severity of tubal factor infertility (TFI; 0 = no occlusion, 1 = unilateral occlusion, 2 = bilateral occlusion).

**Figure 2.**
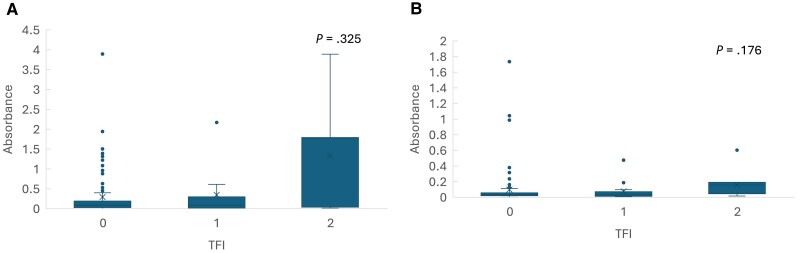
The absorbance values of *Chlamydia trachomatis* and Hsp60 IgG1 (*A*) and IgG3 (*B*) antibody according to the severity of tubal factor infertility (TFI; 0 = no occlusion, 1 = unilateral occlusion, 2 = bilateral occlusion.

Pgp3 IgG1 and IgG3 absorbances increased with increasing severity of tubal damage (mean absorbance 0.63 in non-TFI, 1.26 in unilateral tubal occlusion, and 2.67 in bilateral tubal occlusion for Pgp3 IgG1, *P* = .001; 0.16, 0.35, and 2.01 for Pgp3 IgG3, *P* = .012, respectively) (Figure 1). Hsp60 absorbance also increased by severity of tubal damage, but the differences were not significant (*P* = .33 for Hps60 IgG1 and *P* = .18 for IgG3) (Figure 2).

Table 3 shows the performance of *C. trachomatis* Pgp3 IgG1 and IgG3, and Hps60 IgG1 and IgG3 in detecting tubal pathology. Pgp3 IgG1 antibody measurement was the most sensitive (68.2%; 95% CI, 45.1%–86.1%) and Hsp60 IgG3 the most specific (93.6%; 95% CI, 89.7%–96.4%) for TFI.

**Table 3.** The Predictive Value of *Chlamydia trachomatis* Pgp3 and Hsp60 IgG Antibodies in the Prediction of Tubal Factor Infertility

Antibody	Sensitivity, % (95% CI)	Specificity, % (95% CI)	Accuracy, % (95% CI)	PPV, % (95% CI)	NPV, % (95% CI)	LR Positive (95% CI)	LR Negative (95% CI)
Pgp3 IgG1	68.2 (45.1–86.1)	68.2 (61.9–74.1)	68.2 (62.2–73.9)	16.7 (12.5–22.0)	95.8 (92.5–97.7)	2.2 (1.5–3.0)	0.5 (.3–.9)
Pgp3 IgG3	40.9 (20.7–63.7)	85.2 (80.0–89.5)	81.4 (45.1–86.1)	20.5 (12.5–31.6)	93.9 (91.6–95.7)	2.8 (1.5–5.0)	0.7 (.5–1.0)
cHSP60 IgG1	36.3 (17.2–59.3)	83.1 (77.6–87.6)	79.1 (73.6–83.9)	16.7 (9.7–27.1)	93.3 (91.0–95.1)	2.2 (1.2–4.0)	0.8 (.6–1.1)
cHSP60 IgG3	22.7 (7.8–45.4)	93.6 (89.7–86.4)	87.6 (82.991.4)	25.0 (11.8–45.4)	92.9 (91.2–94.2)	3.6 (1.4–8.9)	0.8 (.7–1.0]

Abbreviations: CI, confidence interval; LR, likelihood ratio; NPV, negative predictive value; PPV, positive predictive value.

### *C. trachomatis* Pgp3 and Hsp60 IgG1 and IgG3 Antibodies in NAAT-Positive Women

A total of 26 of 33 (78.8%) *C. trachomatis* NAAT-positive women were seropositive for *C. trachomatis* Pgp3 IgG1, and 24 (72.7%) for Pgp3 IgG3. However, 6 women had neither IgG1 nor IgG3 Pgp3. Among seropositives, the mean absorbance value of Pgp3 IgG1 was 3.17 (1.1; 0.3–4), and 2.31 (1.4; 0.2–4) of Pgp3 IgG3. Hsp60 IgG1 antibody was present in 18 (54.5%) and IgG3 in 6 (18.2%) of the *C. trachomatis* NAAT-positive women.

## DISCUSSION

We found that Pgp3 IgG1 and IgG3 antibodies were strongly associated with TFI among subfertile women. Hsp60 IgG1 and IgG3 were also linked to TFI, but the overall prevalence was lower. Ascending *C. trachomatis* infection damages fallopian tubes and causes pelvic adhesions, particularly after untreated or repeat chlamydial infection [2, 25, 26]. The estimated risk of TFI is up to 6.9% after 1 episode of *C. trachomatis*-induced PID, and it increases after repeat infections [2, 27, 28]. The Pgp3 protein may play a significant role in the pathogenesis of *C. trachomatis* infection, as evidenced by less pathology associated with infections caused by plasmid-free strains [29]. Pgp3 has been implicated in long-lasting inflammation and tubal scarring [30].

We found that the *C. trachomatis* Pgp3 IgG1 response was superior in detecting prior self-reported *C. trachomatis* infection among subfertile women, outperforming other serological markers such as MOMP, TroA, and HtrA IgG [5, 6, 23, 31]. This is in line with prior studies showing that Pgp3 IgG is an accurate serological marker of *C. trachomatis* exposure, with antibodies persisting more than 10 years in women [11, 12]. However, it is important to measure IgG1 and IgG3 antibodies separately, as IgG1 may reflect past infection due to its longer lifespan, while IgG3 antibody may reflect more recent infection [21]. Our results are in agreement with this, as Pgp3 IgG3 antibody was more frequently detected in sera from NAAT-positive women (78.8%) than from subfertile women (17.1%) or from women with TFI (40.9%).

When evaluating diagnostic accuracy in TFI screening, several challenges exist. Predictive value of a test depends on performance of the laboratory assay, definition of TFI, and the reference group used. Conventional *C. trachomatis* serology predicts TFI poorly, mainly due to a high rate of false-positive cases, leading to a low positive predictive value [5, 23, 32]. In our study, while Pgp3 IgG1 was the most sensitive test, its specificity was only 68.2%, due to a high number of seropositive cases among controls. This may be because not all women exposed to *C. trachomatis* develop TFI, and Pgp3 IgG1 may become positive after a single infection, which relatively rarely leads to TFI. Serum antibodies may diminish over time in those with asymptomatic, uncomplicated infection compared to those with repeated infection or reproductive sequelae [33, 34]. We found that Pgp3 IgG3 had higher specificity and accuracy in predicting TFI, although lower sensitivity due to the short-lived nature of IgG3. For screening purposes, however, sensitivity is generally considered more important than specificity.

Chlamydial Hsp60 has been linked to chronic *C. trachomatis* infection, and serum antibody to Hsp60 has been associated with TFI [35–37]. We also found Hsp60 antibodies more often in women with TFI than in women with non-TFI. However, the overall prevalence of chlamydial Hsp60 antibodies was low, suggesting that the presence of these antibodies is not a strong marker of scarring after *C. trachomatis* infection-induced inflammation. While Hsp60 IgG3 showed high specificity (92.9%) for TFI, sensitivity was low (25.0%). Recently, *C. trachomatis* infection-associated induction of growth factor signaling and profibrotic remodeling of the extracellular matrix have been proposed as mediators of scarring [15]. Whether Hsp60 plays a role in the proinflammatory stimulation of infected tissues facilitating the development of fibrosis remains to be elucidated.

Among women NAAT-positive for *C. trachomatis*, 82% had serum Pgp3 IgG antibodies (either IgG1 or IgG3) in a serum sample taken on the same day as that of the patient's positive NAAT result. The Pgp3 seropositivity was high but comparable to other studies using indirect ELISA [10, 12, 38]. The rather high positivity rate suggests that many individuals had repeat infections [38], but this could not be confirmed. Cross-reactivity with antibodies to *C. pneumoniae* is unlikely to explain the high seropositivity rate [10, 11]. The observed elevated antibody levels are consistent with a previous report using similar ELISA [12]. Those who remained seronegative may not have developed detectable antibody response either due to passive infection [2], early course of the infection [39], or infection caused by plasmid-free *C. trachomatis* [40].

The double-antigen Pgp3 ELISA is a highly sensitive assay and less likely to lose sensitivity over time [38]. While our study did not directly compare the performance of the Pgp3 IgG1 ELISA with the double-antigen Pgp3 ELISA, our results demonstrated a mean absorbance value of Pgp3 IgG1 among *C. trachomatis* NAAT-positive and seropositive women as high as 3.17, with a cutoff absorbance value of just 0.263. In comparison, a previous study reported a cutoff absorbance value of 0.44 for the double-antigen Pgp3 assay [11]. Furthermore, Pgp3 IgG1 absorbance values correlated with the severity of tubal damage, with mean absorbance values of 0.63 in non-TFI, 1.26 in unilateral tubal occlusion, and 2.67 in bilateral tubal occlusion (*P* = .001). This suggests that Pgp3 IgG1 levels can serve as a predictor for the severity of tubal damage. These findings indicate that the Pgp3 IgG1 ELISA used in this study is a highly sensitive assay, capable of maintaining Pgp3 seropositivity over time, and potentially comparable or even superior to the double-antigen Pgp3 ELISA.

Our study had limitations such as small number of TFI cases and inability to study antibody persistence over time. Nonetheless, the strengths include consistent eligibility criteria, outcome measures, and testing of IgG subclasses.

In summary, we studied IgG1 and IgG3 antibody responses to 2 *C. trachomatis* proteins, Pgp3 and Hsp60, among infertile women with TFI. We found that IgG antibody to Pgp3 was a sensitive marker for ongoing or past *C. trachomatis* infection. Pgp3 IgG1 antibody was the most sensitive (68.2%) marker of TFI. This is in line with the fact that TFI can also be caused by other factors. Hsp60 IgG3 was the most specific marker for TFI (92.9%). Further exploration of additional chlamydial antigens, such as TroA, HtrA [5], and MOMP [23], using this approach is important. Combining multiple chlamydial antigens and antibody isotypes may offer a sensitive and specific method for diagnosing TFI.

## Notes

***Acknowledgment.*** We thank Anu Kaitonen for excellent technical assistance.

***Financial support.*** This work was supported by Helsinki University Hospital research funding (grant number TYH2023329 to M. P.).
